# Functional genome analysis reveals that serine carboxypeptidase *Bd-SCP10* mediates vegetative growth, pathogenicity, and stress tolerance in *Botryosphaeria dothidea*

**DOI:** 10.3389/fpls.2025.1678786

**Published:** 2025-11-14

**Authors:** Muhammad Umer, Naureen Anwar, Mustansar Mubeen, Yun Li, Khalid M. Alsyaad, Ahmed Ezzat Ahmed, Pingwu Liu

**Affiliations:** 1 School of Breeding and Multiplication (Sanya Institute of Breeding and Multiplication), College of Tropical Agriculture and Forestry, Hainan University, Sanya, Hainan, China; 2 Department of Biological Sciences, Faculty of Science and Technology, Virtual University, Lahore, Pakistan; 3 Department of Plant Pathology, College of Agriculture, University of Sargodha, Sargodha, Pakistan; 4 College of Agriculture, Guangxi University, Nanning, China; 5 Department of Biology, College of Science, King Khalid University, Abha, Saudi Arabia; 6 Prince Sultan Bin Abdelaziz for Environmental Research and Natural Resources Sustainability Center, King Khalid University, Abha, Saudi Arabia

**Keywords:** *Botryosphaeria dothidea*, eco-friendly disease control strategies, functional genomics, pathogenicity, serine carboxypeptidase S10 family

## Abstract

**Introduction:**

*Botryosphaeria dothidea* (*B*. *dothidea*) is a catastrophic fungal pathogen that threatens fruit production worldwide. Secreted peptidases like serine carboxypeptidases (*SCPs*) are well known to be involved in fungal virulence, but their role in *B. dothidea* is unknown.

**Methodology:**

Here, we identified and functionally characterized *Bd-SCP10*, a homolog of *SCPs* found in *B. dothidea*, which is a member of the S10 family, using a split marker strategy for gene knockout and complementation.

**Results:**

Mutants exhibited substantial phenotypic changes, including reduced radial growth and compromised biomass production, as well as altered pathogenicity and stress tolerance in response to multiple stress conditions. In contrast, complementation restored these traits, suggesting a functional role of *Bd-SCP10*. Particularly, *Bd-SCP10* contributes to maintaining growth, cell wall integrity and adaptation to host-induced stresses, highlighting its involvement in fungal survival and pathogenicity.

**Discussion:**

This study provides the first functional evidence that secreted peptidases in *B. dothidea* are a key factor in vegetative growth, pathogenicity, and stress tolerance. The identification and functional characterization of *Bd-SCP10* led us to believe that it is a promising molecular target for eco-friendly strategies to manage diseases caused by *B. dothidea* and related pathogens.

## 1 Introduction

*B*. *dothidea* infects many woody plants worldwide and damages most parts of plants, including leaves, fruits, branches, and stems. It can cause fruit rot, leaf spot, twig dieback, and stem and branch canker diseases, leading to the death of infected trees (Jash et al., 2025). It has caught the attention of fruit tree pathologists, as it can infect most economically significant fruit trees, such as apples, pears, grapes, peaches, and blueberries, and cause severe diseases resulting in substantial annual losses. In China, apple white rot caused by *B. dothidea is* considered as one of the most destructive diseases (Dong and Guo, 2020). Raindrops facilitated the spread of fungal conidia to the host tissue surface, and conidia primarily form a germ tube and appressorium. After the development of the appressoria appressorium, it begins to invade cells and expand inside the cell, then infects the cells and absorbs nutrients from the infected tissues (Kim et al., 2016). Eco-friendly strategies for disease control measures are still unknown for this devastating fungal pathogen. That is why it is necessary to explore sustainable approaches. The host defense system is considered the first line of defense against fungal invasion, and the plant cell wall can provide an adequate defense against fungal attacks (Looi et al., 2017; Munzert and Engelsdorf, 2025). Alternatively, fungal pathogens employ a range of strategies, including physical, enzymatic (such as hemicellulase, cutinase, pectinase, cellulase, lipases, secreted peptidases, and degradative enzymes), and chemical effectors to overcome barriers during invasion and infection (Zhao et al., 2013; Rauwane et al., 2020). Comparative genomic analysis of *B. dothidea* has suggested that virulence- and pathogenicity-related genes are involved in regulating metabolic pathways associated with the production of various enzymes, including those involved in plant cell wall degradation, biosynthesis, cytochrome P450, carbohydrate-active, and secreted peptidases (Wang et al., 2018). Plant cell wall degrading and secreted peptidases are considered the most important factors related to pathogenicity, and the former have been well characterized as responsible for virulence in many phytopathogenic fungi. While the latter remains enigmatic (Wang et al., 2018; Munzert and Engelsdorf, 2025). *SCPs* are proteolytic enzymes belonging to the peptidase S10 family, characterized by a conserved Ser–Asp–His catalytic triad (Oda et al., 2022) and hydrolyze peptide bonds from the C-terminus of peptides and proteins (Semenova et al., 2020), thereby contributing to protein processing, nutrient acquisition, and regulation of cellular functions (Chen et al., 2022). In fungi, *SCPs* are often secreted into the extracellular environment, where they interact with host proteins and cell wall components, facilitating the release of nutrients and promoting host colonization. Beyond their housekeeping functions, *SCPs* are increasingly recognized as essential virulence factors (Muszewska et al., 2017; Da Silva, 2018; Madhu et al., 2020). They vary in their characteristics, including substrate selectivity, active site, and catalytic mechanism, and are also involved in a wide range of complex physiological activities (Da Silva et al., 2006; Dong et al., 2021). Generally, fungal pathogens secrete peptidases to modify spore production and germination and these enzymes act as virulence regulators (Krishnan et al., 2018; Yang et al., 2023). In addition, fungal peptidases can inhibit the host defense system by modifying and inactivating protein components of the protection machinery of the host (Xia, 2004; Shi et al., 2025). *Fusarium graminearum*, *FgSCP* was shown to be essential for fungal growth, toxin biosynthesis, stress tolerance, pathogenicity, and suppression of host immunity (Liu et al., 2024). Similarly, in *Penicillium expansum*, secretome profiling identified a *PeBgl1* and *PeSCP* as enzymes required for virulence on apple fruit, emphasizing their direct role in host invasion (Wang et al., 2025). Several old studies have also shown the importance of secreted peptidases in the regulation of virulence in fungal pathogens, e.g., *SsNEP2* in *Sclerotinia sclerotiorum* (Yang et al., 2022), *BcCGF1* in *Botrytis cinerea* (Zhang et al., 2020a), *CpSge1* in *Cryphonectria parasitica* (Lin et al., 2025b), *Fospc2* in *Fusarium odoratissimum* (Yang et al., 2023), and *GcStuA* in *Glomerella cingulate* (Chethana et al., 2021). These findings highlight the functional role of *SCP*s as key modulators of fungal physiology, managing growth, stress adaptation, and host immune suppression. Although the recognized roles of *SCPs* in other phytopathogenic fungi are well understood, their functional role in *B. dothidea* remains largely unknown. Therefore, identifying the function of *Bd-SCP10* offers a key step toward understanding the mechanisms of pathogenicity and will help in developing innovative, eco-friendly strategies for disease control in fruit crops.

## 2 Materials and methods

### 2.1 Strains and cultural conditions

The *B. dothidea* wildtype strain (Bd-wt) was generously provided by Hafiz Husnain Nawaz (Hainan University, Haikou, China). For colony morphology analysis, the control [CK, representing uninoculated potato dextrose agar medium (PDA)] and Bd-wt were cultured and replicated 6 times on PDA for 5 days (d) at 25°C (Wei et al., 2019).

### 2.2 Nucleic acid manipulations and polymerase chain reaction

Fungal gDNA was isolated from 5 d old mycelium through the 2X Cetyltrimethylammonium bromide (CTAB) method (Jasim et al., 2024). The verification and identification of mutants and complementary strains were performed as previously described through polymerase chain reaction (PCR) (Huang et al., 2017a).

### 2.3 Bioinformatics analysis

The full-length nucleotide sequence of the *Bd-SCP10* gene, along with upstream and downstream flanking sequences, was downloaded from *B. dothidea* (ASM1106463v1, GenBank: GCA_011064635.1, https://www.ncbi.nlm.nih.gov/assembly/GCA_011064635.1/). Oligo primer analysis v. 7.0 (Molecular Biology Insights, Inc., Colorado, USA) was used to design the primers. For phylogenetic analysis, the protein sequence of *Bd-SCP10* (GenBank: KAF4310043.1) was obtained from NCBI, and the homologs of *SCPs* in 12 other phytopathogenic fungi were identified via MEROPS (https://www.ebi.ac.uk/Tools/services/web/toolresult.ebi?jobId=ncbiblast-I20210421-144041-0569-47522791-p2m) (Rawlings, 2020), followed by the multiple sequence alignment with Clustal Omega (https://www.ebi.ac.uk/Tools/msa/clustalo/). Finally, the phylogenetic tree was generated using MEGA software v. 7.0 (Kumar et al., 2016; Ahmad et al., 2021) with the neighbor-joining (NJ) method. The dendrograms were based on clustered homologs, determined through bootstrap analysis (with 1000 replicates), and displayed replication percentages on the respective branches. ORFFINDER (https://www.ncbi.nlm.nih.gov/orffinder/) was used for the analysis of the open reading frame (ORF) (Varabyou et al., 2023), and the protein domain was predicted using Pfam (https://pfam.xfam.org/) (Savojardo et al., 2021).

### 2.4 Targeted knockout of the *Bd*-*SCP10* gene from a wildtype strain

The deletion of the *Bd-SCP10* gene was conducted using a split marker strategy, in which the selectable marker HYG replaced the targeted gene (Yuan et al., 2023). Briefly, in the first round of PCR, the fragment of the upstream flanking sequence (UPS) of *Bd-SCP10* (887 base pairs (bp) was amplified with Up-F and Up-R primers, while the downstream flanking sequence (DNS) of 789 bp was amplified with Dn-F and Dn-R primers. On the other hand, the pCX62 plasmid (Lin et al., 2025a) was used to amplify the HYG resistance cassette for a 767 bp HY fragment with HYG-F/HY-R primers and a 931 bp YG fragment with HYG-R/YG-F primers. In the second round or fusion PCR, the Up-F/HY-R primers were used to fuse the UPS fragment with the HY fragment, while the Dn-R/YG-F primers were used to fuse the DNS fragment with the YG fragment through splicing of overlapped sequence extension, and templates were used in equal proportion in fusion PCR. Fusion PCR cycling conditions were conducted initially at 95 °C for 5 minutes (min), followed by 35 cycles of 95 °C for 30 seconds (s), 60 °C for 45 s, and 72 °C for 90 s, and finally at 72 °C for 10 min. The products of fusion PCR were transformed through the PEG-mediated transformation method (Zhang et al., 2022) into the protoplasts (2×10^7^ cells/mL) isolated from 5 d old mycelium of Bd-wt using the previously described method (Ning et al., 2022). The transformants were cultured on PDA supplemented with 50 µg/mL hygromycin B (HYG; Roche, USA), and the developed colonies were randomly selected and verified by standard PCR using the two sets of primers *Bd-SCP10*-F/*Bd-SCP10*-R and HYG-F/HYG-R.

### 2.5 Complementation of the *Bd*-*SCP10* gene

The complementation of the mutant *ΔBd-SCP10a* was achieved by inserting the Bd-SCP10 gene, along with a NEO resistance cassette, into the knockout mutant through a split marker strategy (Gravelat et al., 2012). The first-round PCR was briefly conducted to amplify the 47 bp upstream flanking region (UPS) together with the full length of the *Bd-SCP10* gene, resulting in a 2800 bp amplified fragment and the 832 bp amplified fragment of the downstream flanking region (DNS). Then, the pSELECT-neo plasmid (InvivoGen, USA) was used to amplify the NEO drug cassette for the NE fragment (975 bp) with NEO-F/NE-R primers and the EO fragment (543 bp) with NEO-R/EO-F primers. In the second round or fusion PCR, the cycling conditions were kept the same as those used for deleting the targeted gene. The corresponding primers UP-F/NE-R were used for the fusion of the UPS fragment with the NE fragment. In contrast, the Dn-R/EO-F primers fused the DNS fragment with the EO fragment by splicing an overlapped extension. The products of fusion PCR were transformed through the PEG-mediated transformation method (Zhang et al., 2022) into the protoplasts (1×10^5^ cells/mL) isolated from 5 d old mycelium of mutant *ΔBd-SCP10*a using the previously described method (Ning et al., 2022). Finally, the complementary transformants were screened on PDA supplemented with 150 μg/mL of the antibiotic G418, and the generated colonies were randomly selected and verified by standard PCR using the two sets of primers *Bd-SCP10*-F/*Bd-SCP10*-R and NEO-F/NEO-R.

### 2.6 Phenotype analysis

For the growth rate analysis, mycelial plugs (5 mm diameter) excised from colony margins were placed in the center of PDA (at least 3 replicates) and then incubated for 5 d at 25 °C in the dark (Wei et al., 2019). The per day radial growth rate (Oledibe et al., 2023) was calculated with formula [(Colony diameter – Plug diameter ÷ 2) × Number of incubation days] and per day biomass production rate (Novotna et al., 2023) was calculated with formula (Colony weight ÷ Number of incubation days) and final measurements were taken at 5 days post-inoculation (dpi). The hyphal tips of fungal strains were observed under a Ni90 microscope (Nikon, Japan) after 5 dpi incubation, following the previous method (Aboelfotoh Hendy et al., 2019; Zhang et al., 2020b).

### 2.7 Virulence assay

Virulence assay was performed on pear fruits (*Pyrus bretschneideri* var. huangguan) with the mycelial plugs (5 mm diameter) derived from actively growing colonies (at least 6 replicates), and uncolonized PDA plugs were involved in parallel as a control (CK). Mechanically produced wounds with a sterile needle on the fruits were used for inoculation by reversely placing mycelial plugs. After inoculation, the fruits were incubated at 25 °C with 100% relative humidity. The resulting lesions were measured at 5 days post-infection (dpi), and photos were taken to document lesion development.

### 2.8 Stress response assays

For a stress response assays, mycelia of Bd-wt, complementary strains, and mutants were quantitatively analyzed after cultured on unamended PDA or amended PDA [supplemented with different stress agents (at least 6 replicates), including 0.04% sodium dodecyl sulfate (SDS), 0.5 M calcium chloride (CaCl_2_), 1 M glucose (C_6_H_12_O_6_), 1.5 M sodium chloride (NaCl), 1 M potassium chloride (KCl), and 0.05% hydrogen peroxide (H_2_O_2_)] for 5 d, respectively. The percentage growth inhibition rates were calculated with the formula [(Do – Dt ÷ Do) × 100], where Do represents the mean colony diameter on PDA, and Dt represents the mean colony diameter on amended PDA (Khanh et al., 2024).

### 2.9 Statistical analysis

The quantitative data were statistically analyzed using Statistix v. 8.1 (Analytical Software, Florida, USA) with ANOVA and the least significant difference (LSD) test at *P ≤ 0.05*. The bar graphs were drawn on GraphPad Prism v. 8.0 (GraphPad Software, California, USA).

## 3 Results

### 3.1 Phenotypic and virulence assay of the *B. dothidea* wildtype strain

For phenotypic analysis, the CK and Bd-wt were cultured on PDA for 5 d at 25 °C. CK culture showed a plain dish of PDA without fungal growth, while Bd-wt culture showed the whitish color of the colony with a fluffy cotton-like white margin and a relatively thick mycelium mat, as shown in **Supplementary Figure S1A**. For virulence assays, CK does not induce disease on pear fruits (var. huangguan, 6 replicates). However, lesions induced by Bd- wt can be seen in **Supplementary Figure S1B** after 5 dpi. Therefore, these results suggested that Bd-wt is a virulent strain.

### 3.2 Identification and characterization of the *Bd*-*SCP10* gene

**Figure 1:**
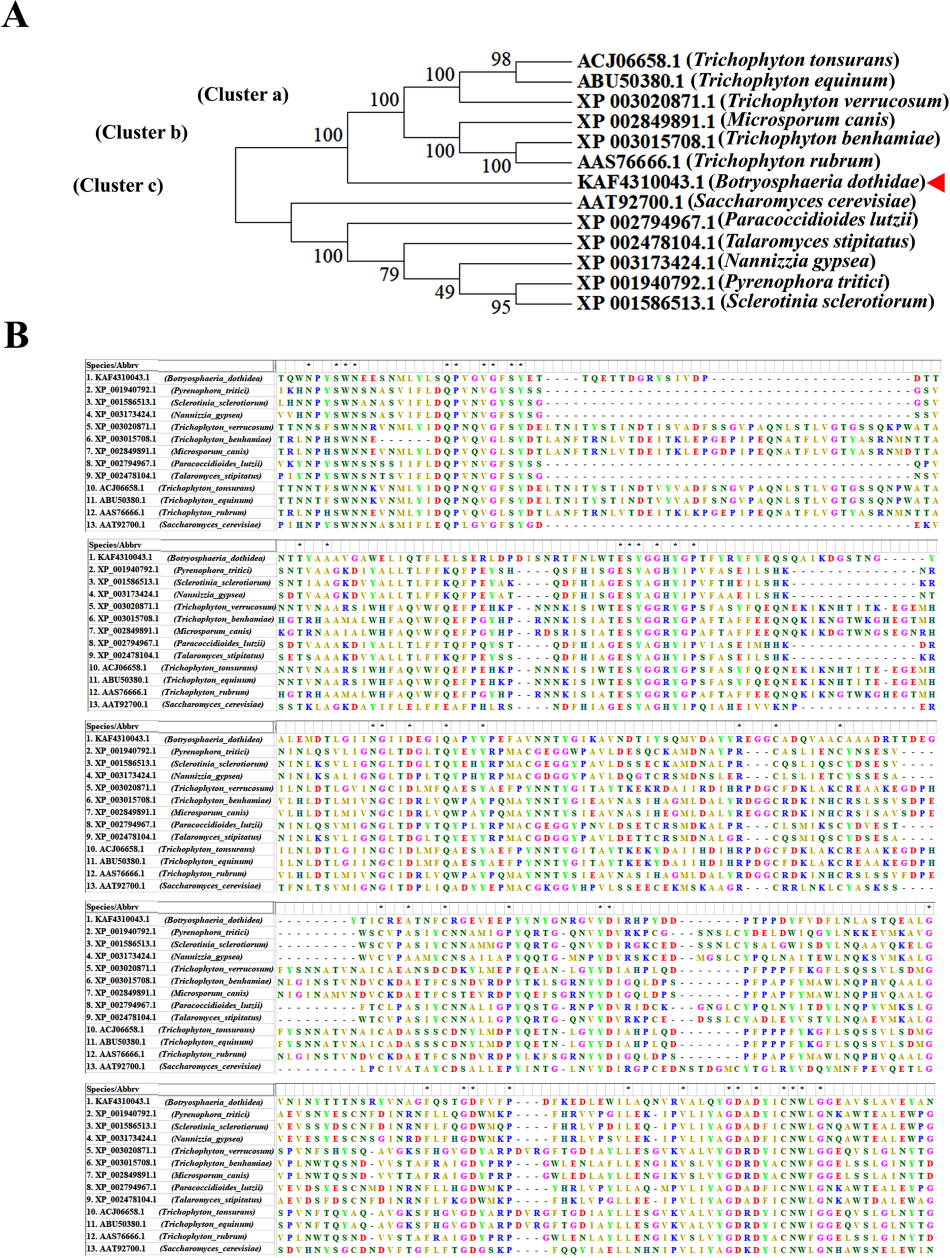
Phylogenetic analysis and multiple sequence alignment of *Bd-SCP10* together with other fungal homologs of *SCPs.***(A)** The phylogenetic tree was generated using MEGA v. 7.0 with the neighbor-joining method, and fungal *SCPs* were grouped into 3 clusters (a to c) as indicated by the arrow. The number beside the branch nodes refers to the bootstrap value, and the host’s fungal name is presented in brackets, followed by the homolog accession number. *B. dothidea* is indicated with a triangle ahead of its accession number. **(B)** The sequences of amino acids are aligned using Clustal Omega, and conserved amino acids among the selected pathogenic fungi are denoted by an asterisk (*).

The MEROPS database analysis of amino acid sequences similar to *SCPs* belonging to the peptidase S10 family revealed its homolog in *B. dothidea*, which was tentatively named as *Bd-SCP10*. *Bd-SCP10* shared the highest identity (40.9%, *E-value* = 8.9E^-133^) with the homolog in *Microsporum canis* (EEQ30006.1), a member of the family Arthrodermataceae. A phylogenetic tree was constructed based on the amino acid sequences of *Bd-SCP10* together with other homologs of *SCPs* chosen from 12 fungal species based on percentage amino acid sequence identity and *E-value* (32% to 40.9%, and 8.9E^-133^ to 7.9E^-57^, respectively). The neighbor-joining phylogenetic tree showed that all the fungal *SCPs* were grouped into 3 clusters (a to c), and *Bd-SCP10* was located in a separate clade far from other members in cluster A and that has been shown in **Figure 1A**. The multiple sequence alignment results depicted that *Bd-SCP10* belongs to the peptidase S10 family (PF00450) and this family have 43 conserved amino acids among in all of the selected phytopathogenic fungi shown in **Figure 1B**.

### 3.3 Knockout of the *Bd*-*SCP10* gene from a wildtype strain

**Figure 2:**
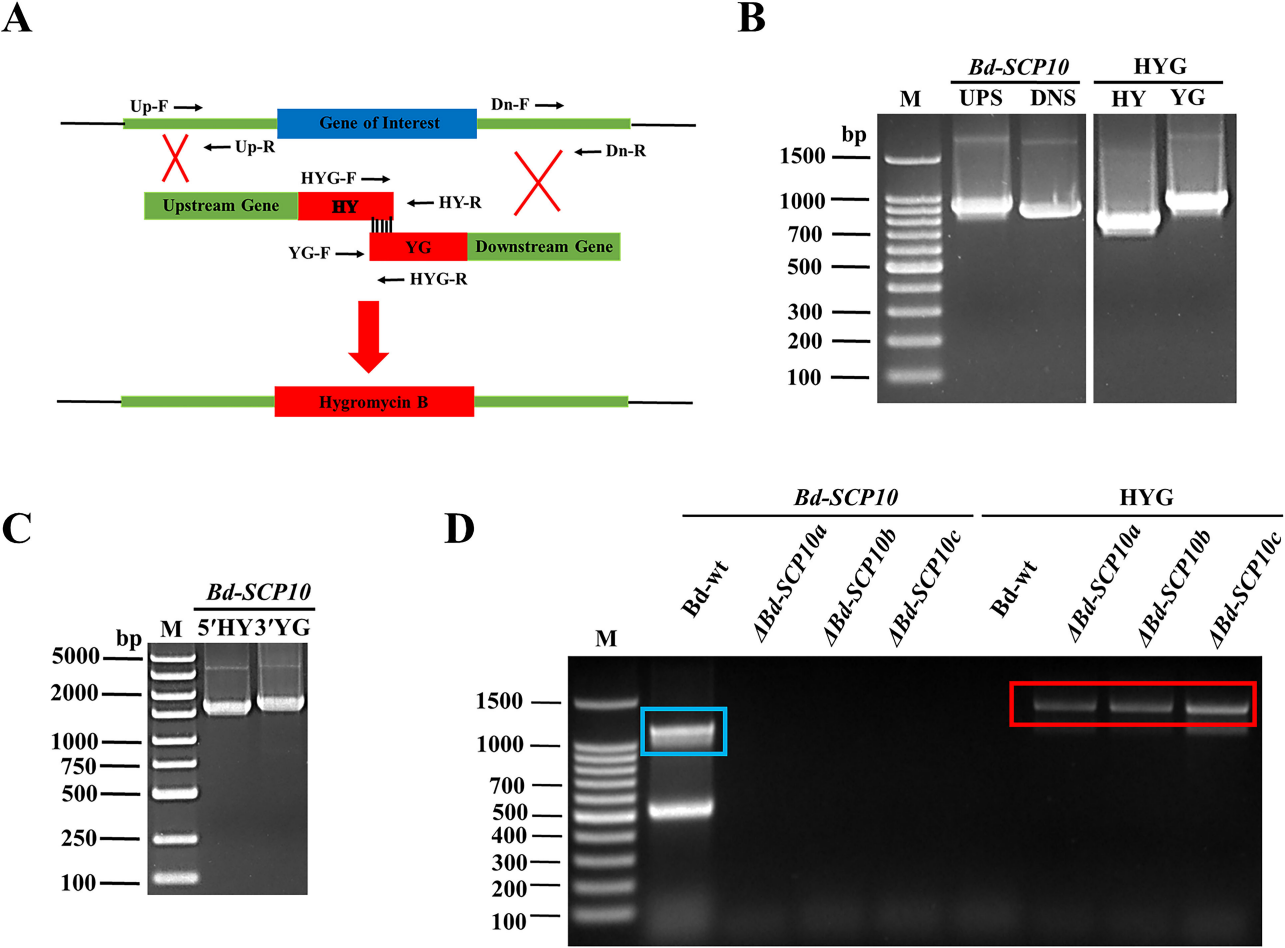
Targeted knockout of *Bd-SCP10* from *B. dothidea* through split marker strategy and PCR identification of the mutants. **(A)** Schematic representation of the split marker strategy for deleting *Bd-SCP10* from Bd-wt. **(B)** First-round PCR amplified fragments (UPS: 887 bp, DNS: 789 bp, HY: 767 bp, and YG: 931 bp). **(C)** Fusion PCR products showing the synthesis of the two recombinant fragments 5′HY (1654 bp) and 3′YG (1720 bp) used for transformation. **(D)** PCR identification for the *Bd-SCP10* gene and HYG resistance cassette in Bd-wt and mutants. Amplification of *Bd-SCP10* (blue box, amplified band of 1200 bp) was detected only in Bd-wt, while amplification of the HYG cassette (red box, amplified band of 1383 bp) was detected only in mutants (*ΔBd-SCP10a*, *ΔBd-SCP10b*, and *ΔBd-SCP10c*), confirming successful gene knockout. Here, M is denoted for DNA markers (Note: The original gel documentation figures for **(B, C)** can be seen in **Supplementary Figure S2**).

To verify the gene function of *Bd-SCP10*, Bd-wt induced long lesions (approximately 35.8 mm) as inoculated on pear fruits (*Pyrus bretschneideri* var. huangguan) as shown in **Supplementary Figures S1A, B**, was subjected to knockout for the target gene through split marker strategy, i.e., *Bd-SCP10* gene was replaced with a HYG resistance cassette through homologous recombination (Dong and Guo, 2020) of the flanking parts around the targeted gene and schematic representation has been shown in **Figure 2A**. The first-round PCR was conducted to amplify 887 bp UPS and 789 bp DNS fragments, as well as the HY (767 bp) and YG (931 bp) fragments of the HYG resistance cassette as shown in **Figure 2B** and **Supplementary Figure S2A**. The UPS fragment was further fused with the HY fragment using fusion PCR, generating the fragment termed as 5′HY (1654 bp). Similarly, fragment DNS was fused with YG to generate a fragment termed as 3′YG (1720 bp), which is shown in **Figure 2C** and **Supplementary Figure S2B**. The resulting DNA fragments 5′HY and 3′YG were purified and used to transfect the protoplasts of Bd-wt through PEG-mediated transformation. Upon transfection, a UPS-HYG-DNS combination replaced the *Bd-SCP10* gene through homologous recombination. After transfection, 12 colonies were grown in media containing the antibiotic HYG, and 3 colonies named *ΔBd-SCP10*a*, ΔBd-SCP10*b, and *ΔBd-SCP10*c were selected for identification by a strict PCR strategy with *Bd-SCP10*-F/*Bd-SCP10*-R and HYG-F/HYG-R primers mentioned in **Supplementary Table S1**, targeting *Bd-SCP10* and HYG fragments, respectively. The results showed that the *Bd-SCP10* amplified fragment of 1200 bp was detected in Bd-wt except in mutants, while the HYG amplified fragment of 1383 bp was present in mutants except for Bd-wt, as depicted in **Figure 2D**, suggesting that *Bd-SCP10* was successfully knocked out.

### 3.4 Complementation of the *Bd*-*SCP10* gene

**Figure 3:**
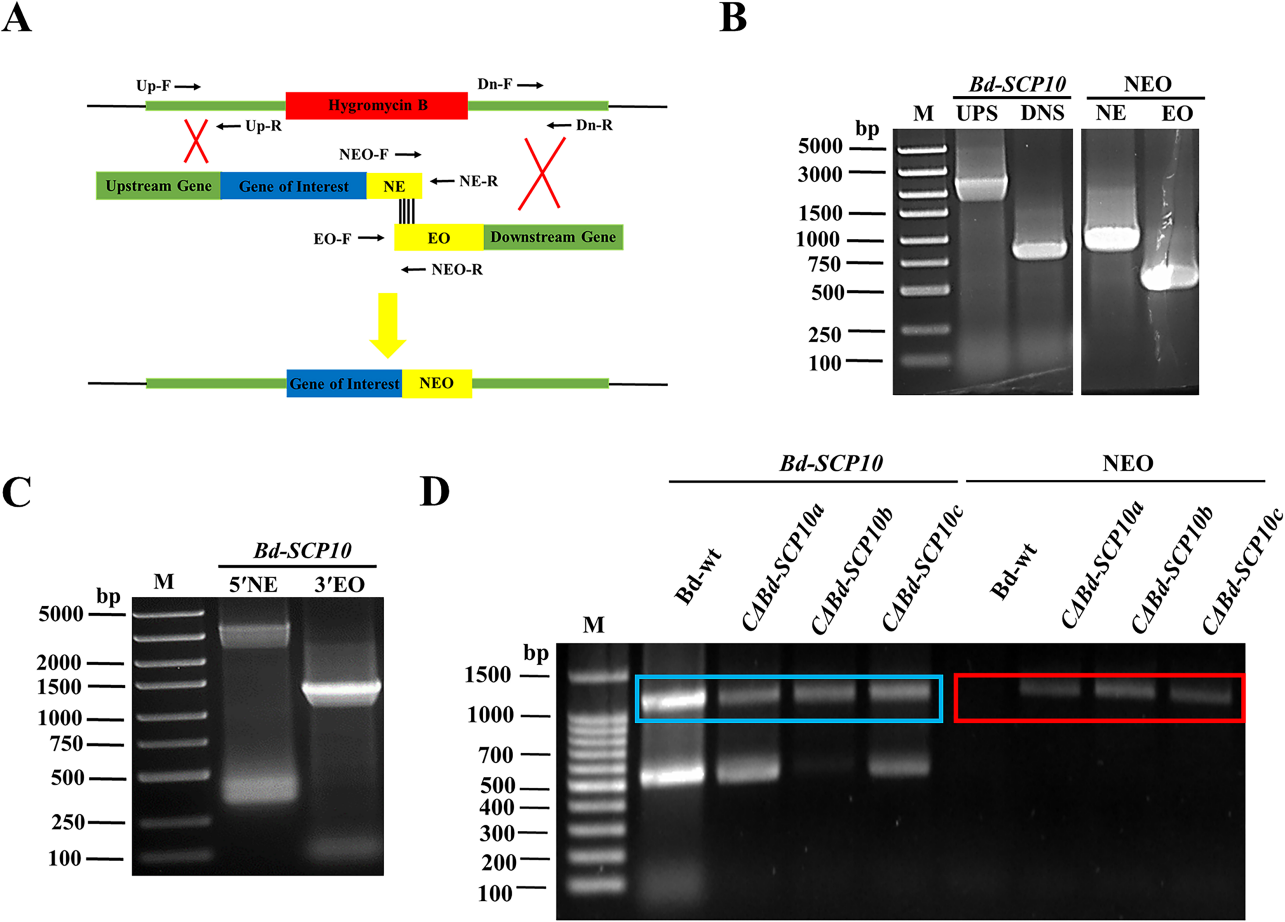
Complementation of the *Bd-SCP10* mutant through the split marker strategy and PCR identification of the complementary strains. **(A)** Schematic representation of the complementation through the split marker strategy, in which *Bd-SCP10* was reintroduced into mutant *ΔBd-SCP10a* with the NEO resistance cassette. **(B)** First-round PCR fragments (UPS: 2800 bp, DNS: 832 bp, NE: 975 bp, and EO: 543 bp). **(C)** Fusion PCR products, which showed recombinant fragments (5′NE, 3775 bp; 3′EO, 1375 bp), were used for transformation. **(D)** PCR identification for the *Bd-SCP10* gene and NEO resistance cassette in Bd-wt and complementary strains. *Bd-SCP10* (blue box, amplified band of 1200 bp) was detected in Bd-wt and complementary strains (*CΔBd-SCP10a*, *CΔBd-SCP10b*, and *CΔBd-SCP10c*), while the NEO cassette (red box, amplified band of 1200 bp) was detected only in complementary strains, confirming restoration of *Bd-SCP10* expression in *ΔBd-SCP10a.* Here, M is denoted for DNA markers (Note: The original gel documentation figures for **(B, C)** are shown in **Supplementary Figure S3**).

The mutant *ΔBd-SCP10*a was subjected for complementation of the targeted gene through split marker strategy, i.e., the HYG resistance cassette was replaced with gene *Bd-SCP10* and NEO resistance cassette through homologous recombination (Dong and Guo, 2020) of the flanking parts around the HYG resistance cassette and schematic representation can been seen in **Figure 3A**. The first-round PCR was conducted to amplify the 2800 bp UPS and 832 bp DNS fragments, along with the NE (975 bp) and EO (543 bp) fragments of the NEO resistance cassette, as shown in **Figure 3B** and **Supplementary Figure S3A**. The UPS fragment was further fused with the NE fragment using fusion PCR, resulting in the 5′NE (3775 bp) fragment. At the same time, the DNS with EO generated the 3′EO (1375 bp) fragment, as depicted in **Figure 3C** and **Supplementary Figure S3B**. The resulting DNA fragments 5′NE and 3′EO were purified and used to transfect the protoplasts of the mutant *ΔBd-SCP10*a through PEG-mediated transformation. Upon transfection, a UPS-NEO-DNS combination replaced the HYG resistance cassette through homologous recombination. After transfection, 8 colonies were grown in the media containing G418 antibiotic, and colonies *CΔBd-SCP10*a*, CΔBd-SCP10*b, and *CΔBd-SCP10*c were picked for identification by PCR strategy with *Bd-SCP10*-F/*Bd-SCP10*-R and NEO-F/NEO-R primers described in **Supplementary Table S1**, targeting *Bd-SCP10* and NEO fragments, respectively. The results showed that the amplified fragment of 1200 bp *Bd-SCP10* was detected in Bd-wt together with complementary strains, while the NEO amplified fragment of 1200 bp was present in complementary strains except for Bd-wt, as shown in **Figure 3D**, suggesting that complementation of *Bd-SCP10* was successful and complementary strains were generated from mutant *ΔBd-SCP10*a.

### 3.5 *Bd*-*SCP10* gene is required for vegetative growth and virulence

**Figure 4:**
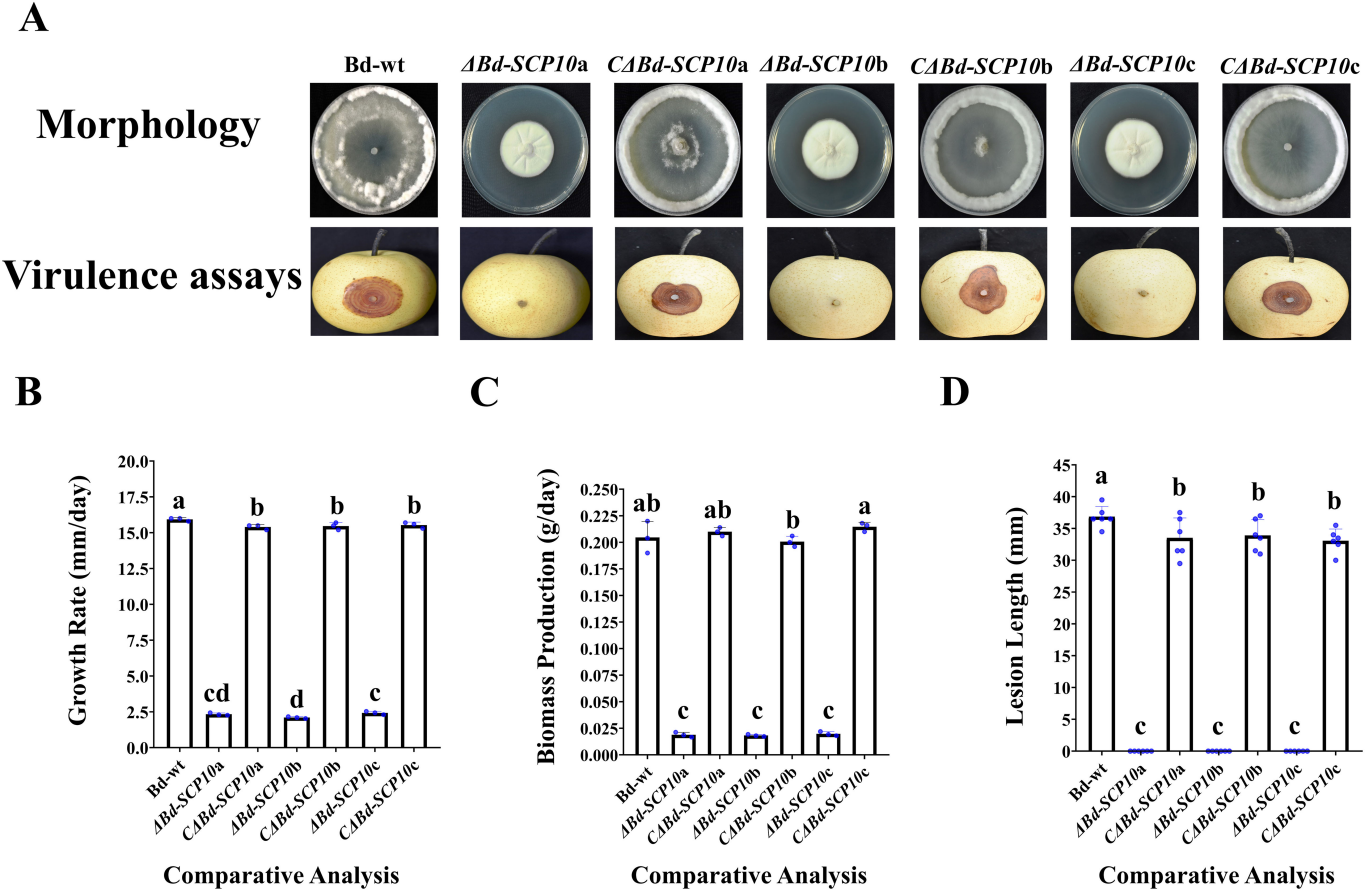
Comparative analysis of Bd-wt, mutants, and complementary strains for vegetative growth, biomass production, and virulence. **(A)** Phenotype and induced symptoms on the inoculated pear fruits (*Pyrus bretschneideri* var. huangguan) for Bd-wt, mutants, and complementary strains. **(B-D)** Bar graphs for the growth rates, biomass, and lesion lengths on the inoculated pear fruits for Bd-wt, mutants, and complementary strains, respectively. The alphabet on a column represented analysis of variance (ANOVA) with the least significant difference (LSD) at *P ≤ 0.05.* During this experimental process, 3 replicates were used for vegetative growth and biomass production, and 6 replicates were used for the virulence assay.

To test whether *Bd-SCP10* is related to the fungal vegetative growth, the mutants and complementary strains, along with Bd-wt, were cultured on PDA in triplicate for 5 d and subjected to assessment of their phenotype, growth, and biomass. These mutants exhibited snowy white and compact aerial mycelium throughout the entire colonies, characterized by random radial grooves. In contrast, both complementary strains and Bd-wt showed a collapsed and cleared mycelial growth in the colony center. At the same time, upward fluffy and cottony mycelia in the margins can be seen in **Figure 4A**. Additionally, the mutants exhibited growth rates ranging from 2.1 mm/d (*ΔBd-SCP10*b) to 2.4 mm/d (*ΔBd-SCP10*c), which were substantially reduced compared to Bd-wt (15.9 mm/d). However, the growth rates for *Bd*-*SCP10* complementary strains ranged from 15.4 mm/d (*CΔBd-SCP10*a) to 15.5 mm/d (*CΔBd-SCP10*c), which are nearly identical to Bd-wt, and are shown in **Figure 4B** and **Supplementary Table S2**. The biomass production for the mutants was observed, ranging from 0.018 g/d (*ΔBd-SCP10*b) to 0.019 g/d (*ΔBd-SCP10*c), which were significantly lower than Bd-wt (0.2 g/d), while complementary strains produced biomass approximately 0.2 g/d (*CΔBd-SCP10*b to *CΔBd-SCP10*c), similar to Bd-wt, as shown in **Figure 4C** and **Supplementary Table S2**. Moreover, the hyphal tips of Bd-wt and complementary strains have fewer septa at hyphal tips and more dense mycelial growth than mutants, as observed in **Supplementary Figures S4A, B**. The results showed that *Bd-SCP10* is involved in the phenotypic development and vegetative growth of *B. dothidea*. To test whether *Bd-SCP10* is associated with fungal pathogenicity, the mutants and complementary strains together with Bd-wt were assessed on the pear fruits (var. huangguan, 6 replicates). At 5 dpi, the *Bd*-*SCP10* complementary strains induced lesions ranging from 28.4 mm (*CΔBd-SCP10*c) to 29.1 mm (*CΔBd-SCP10*b). It is almost similar to the lesion lengths (31.1 mm) induced by Bd-wt. In contrast, the mutants did not induce lesions, as shown in **Figures 4A, D**, and **Supplementary Table S3**. These results suggested that *Bd-SCP10* is responsible for the pathogenicity of *B. dothidea*.

### 3.6 *Bd*-*SCP10* gene plays a role in stress tolerance and cell wall integrity

**Figure 5:**
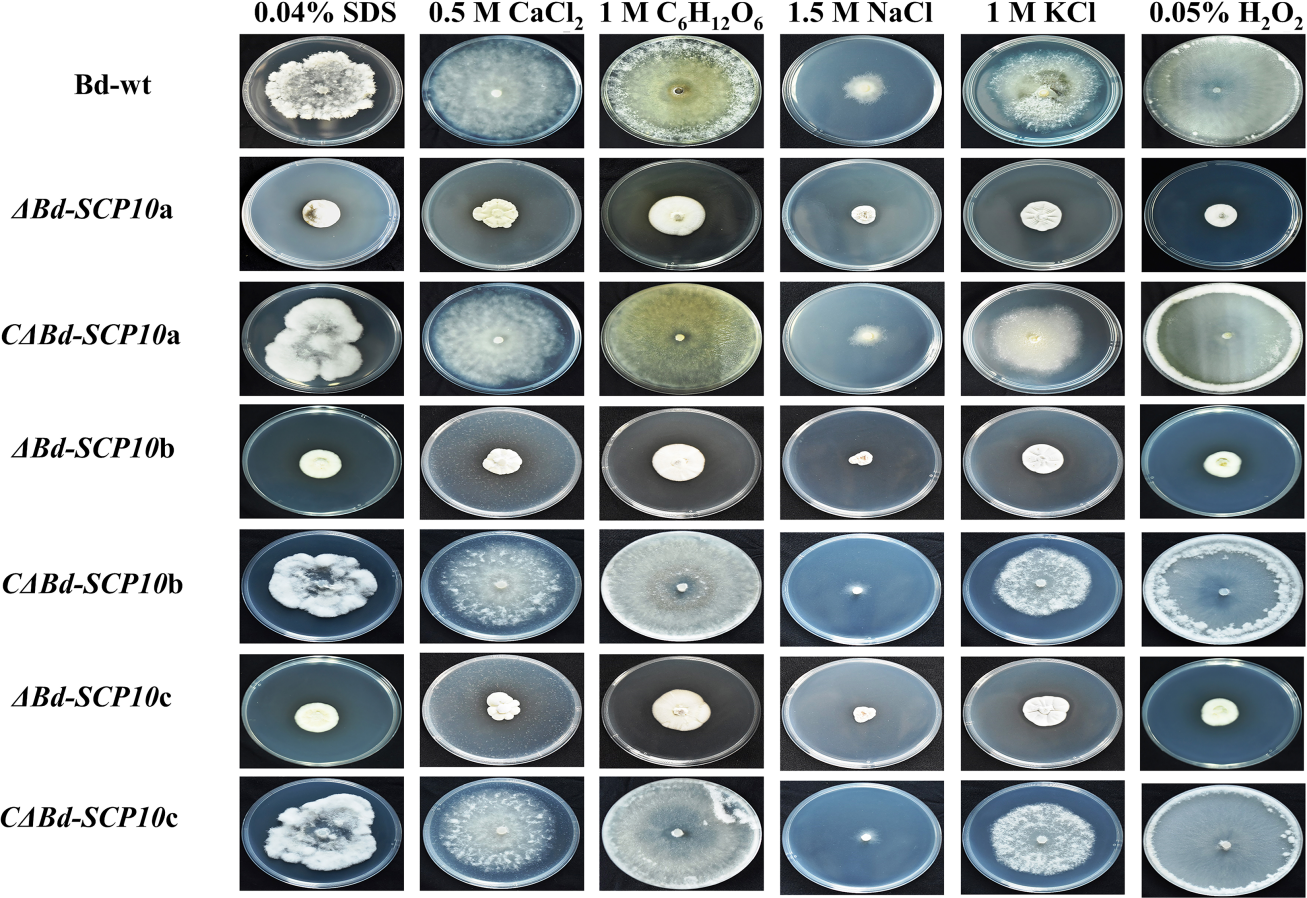
Phenotypic comparison of Bd-wt, mutant, and complementary strains on PDA amended with 0.04% SDS, 0.5 M CaCl_2_, 1 M C_6_H_12_O_6_, 1.5 M NaCl, 1 M KCl, and 0.05% H_2_O_2_ after 5 d at 25 °C. Mutants exhibited reduced growth relative to Bd-wt and complementary strains, particularly under SDS, CaCl_2_, C_6_H_12_O_6_, KCl, and H_2_O_2_ stress.

**Figure 6:**
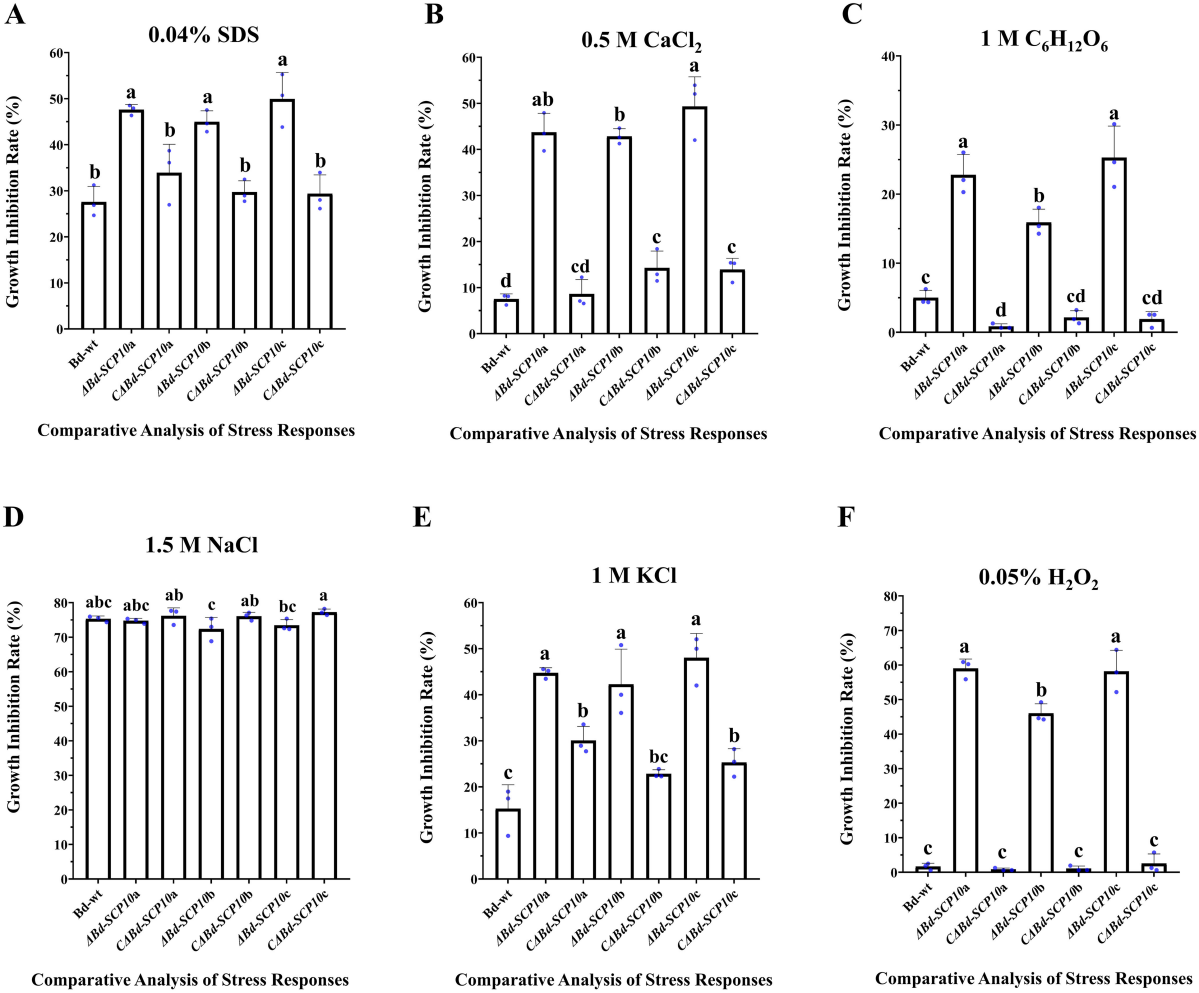
Comparative analysis of stress responses of Bd-wt, mutant, and complementary strains. Growth inhibition rates were measured on PDA amended with 0.04% SDS **(A)**, 0.5 M CaCl_2_**(B)**, 1 M C_6_H_12_O_6_**(C)**, 1.5 M NaCl **(D)**, 1 M KCl **(E)**, and 0.05% H_2_O_2_**(F)**. Alphabet on a columns depicted ANOVA with the LSD at *P ≤ 0.05*, mutants exhibiting pronounced sensitivity to cell wall disrupting, osmotic, and oxidative stresses, underscoring the importance of *Bd-SCP10* in fungal stress tolerance.

Mutants and complementary strains, along with Bd-wt, were cultured on PDA amended with 0.04% SDS to analyze the level of tolerance to cell wall disruption. The results showed significantly higher growth inhibition rates ranging from 45.0% to 49.9% in mutants compared to Bd-wt (27.6%) and complementary strains (29.3% to 33.9%). Similarly, on PDA amended with chemicals used to response against hyperosmotic stresses (0.5 M CaCl_2_, 1 M C_6_H_12_O_6_, 1.5 M NaCl, and 1 M KCl) all mutants showed significantly reduced growth rates on PDA amended with 0.5 M CaCl_2_, 1 M C_6_H_12_O_6_, and 1 M KCl (42.8 to 49.3%, 15.9 to 25.2%, and 42.2 to 48%, respectively) as compared to Bd-wt (7.5%, 5%, and 15.2%) and the complementary strains (8.6 to 14.2%, 15.9 to 25.2%, and 22.8 to 30%). In contrast, the mutants did not show a significant difference in the growth inhibition rates on PDA amended with 1.5 M NaCl (72.4 to 74.7%) as compared with Bd-wt (75.31%) or the complementary strains (76 to 77.2%). Moreover, on PDA amended with 0.05% H_2_O_2_, the response of strains against oxidative stress was observed. The results highlighted that mutant had a significantly higher growth inhibition rate from 46% to 59% than Bd-wt (1.6%) and complementary strains (0.8 to 2.5%), as shown in **Figures 5** and **6**, along with **Supplementary Tables S4** and **S5**. These results suggested that the *Bd-SCP10* gene is involved in cell wall integrity and contributes to the resistance against hyperosmotic and oxidative stresses in *B. dothidea*.

## 4 Discussion

*SCPs* were functionally reported to be involved in protein processing and degradation, as well as the production of secondary metabolites (Chen et al., 2022). This study aligns with emerging evidence that secreted peptidases, particularly *SCPs* belonging to the S10 family, are key virulence factors (Iqbal et al., 2018; Zhang et al., 2019) in phytopathogenic fungi (Semenova et al., 2020). *SCPs* were also identified as potential regulators of phenotypes, habitat adaptation, and growth (Iqbal et al., 2018). However, the functions of *SCPs* in the phytopathogenic fungus *B. dothidea* are still unknown. In this study, we performed functional characterization of the *Bd-SCP10* gene, a homolog of the *SCPs* in *B. dothidea*. Our experimental data confirmed that *Bd-SCP10* plays a key role in vegetative growth, pathogenicity, and stress response, as demonstrated by generating mutants and complementary strains using a split marker strategy. *FgSCP* was previously reported for its role in the virulence of *F*. *graminearum*, and affects fungal growth, stress tolerance, and pathogenicity. It also suppresses cell death triggered by the *INF1* elicitor, indicating its role in modulating plant immune responses (Liu et al., 2024). The substantial reduction in radial growth of Bd-wt (2.1–2.4 mm/d vs. 15.9 mm/d) and biomass production (0.018–0.019 g/d vs. 0.2 g/d) in mutants underscores the importance of the *Bd-SCP10* in fungal physiology. Similar observations were found in *Botrytis cinerea* and *Sclerotinia sclerotiorum*, highlighting that peptidases often facilitate nutrient acquisition by hydrolyzing host proteins into assimilable amino acids (Urbanek and Kaczmarek, 1985; Clark et al., 1997). Moreover, it was reported that *Magnaporthe oryzae* wildtype and complementary strains have denser mycelial development than mutants, as the hyphal tips of the wildtype strain have longer cell lengths and less septation as compared to the mutant, which has more septation and shorter cell lengths (Aboelfotoh Hendy et al., 2019; Zhang et al., 2020b). Our microscopic studies revealed that the thin and compact mycelia of mutants suggested that *Bd-SCP10* regulates hyphal expansion or septation, processes that require precise proteolytic activity to remodel cell walls or recycle proteins (Zhang et al., 2020b). Furthermore, we did not find any differences in colony pigmentation among the mutants, suggesting that the production of secondary metabolites (e.g., melanin or polyketide-derived pigments) was not noticeably affected under PDA culture conditions. Even so, *SCPs* have been reported for the regulation of fungal secondary metabolism in other fungal species (Madhu et al., 2020). It suggested that the *Bd-SCP10* gene is involved in the fungal growth and the phenotypic development of *B. dothidea*. *SCPs* regulate virulence in *Phellinus sulphurascens* (Williams et al., 2014) and *Clonostachys rosea* (Iqbal et al., 2018). A secreted peptidase, BEC1019, regulating a virulence factor, was identified in *Blumeria graminis*, which is required for haustorium development. Silencing of *BEC1019* induced hypovirulence in the fungus (Zhang et al., 2019). An ortholog *Rs-SCP1* was identified in the plant-parasitic nematode *Radopholus similis* and wa*s* involved in reducing pathogenicity (Huang et al., 2017b). Mutations in the *FgSCP* gene compromise nutritional growth and stress tolerance, and deletion leads to reduced pathogenicity in *F*. *graminearum*. Expression is upregulated during infection, suggesting involvement in invasion and potentially by suppressing host defense genes (Liu et al., 2024). The full loss of pathogenicity in mutants on pear fruits and the restoration of pathogenicity in complementary strains represented the essential role of *Bd-SCP10* in infection. Mirror findings in *Fusarium culmorum* revealed that secreted peptidases degrade host defense proteins, enabling tissue colonization by *Fusarium culmorum* (Urbanek and Yirdaw, 1984). Notably, *B*. *dothidea* inoculation relied on wounded fruit surfaces, implying that *Bd-SCP10* acts post penetration by degrading plant cell wall components or suppressing host immune responses. For instance, fungal proteases in *Glomerella cingulata* cleave pathogenesis-related (PR) proteins, neutralizing plant defenses (Clark et al., 1997). The hypersensitivity of *ΔBd-SCP10* mutants to cell wall disrupting agents (0.04% SDS) and hyperosmotic stressors (0.5 M CaCl_2_, 1 M C_6_H_12_O_6_, and 1 M KCl) emphasized the role of *Bd-SCP10* in maintaining cell wall integrity and osmotic homeostasis. These results align with studies related to *Aspergillus fumigatus*, in which *SCPs* were reported to stabilize the cell wall under stress by processing structural proteins (Gravelat et al., 2012). Mannoproteins present in the cell wall of *Candida albicans* play important roles in cell wall remodeling under stress. These proteins interact with cell wall integrity and stress-activated signaling pathways, such as the high-osmolarity glycerol (HOG) and mitogen-activated protein kinase (MAPK) pathways (e.g., Cek1 and Mkc1), which explain how cell wall protein processing ties into stress tolerance (Ibe and Munro, 2021). Although direct evidence connecting *SCPs* to the processing of fungal wall proteins is lacking, the moonlighting capacity of extracellular fungal proteases (Satala et al., 2023) supports the hypothesis that *Bd-SCP10* may serve a functional role in vegetative growth, pathogenicity, and stress tolerance. However, the unaltered sensitivity to 1.5 M NaCl in mutants suggested that *Bd-SCP10* selectively regulates osmotic stress pathways, possibly via interactions with calcium or potassium signaling cascades rather than sodium-specific transporters. In plants, the rapid production and accumulation of reactive oxygen species (ROS) are considered the initial response to invading pathogens (Shetty et al., 2007; Imam et al., 2016). The H_2_O_2_ is a critical ROS that host plants can produce in response to fungal infection and initiate lipid peroxidation, DNA damage, formation of hydroxyl radicals, and protein oxidation (Rolke et al., 2004). Therefore, proper cell wall integrity and stress tolerance are essential for successful fungal infection and thus required for pathogenicity in numerous phytopathogenic fungi (Kim et al., 2009). Similarly, oxidative stress hypersensitivity (0.05% H_2_O_2_) suggests that *Bd-SCP10* may mitigate ROS generated during host invasion, a strategy employed by *Magnaporthe oryzae* to counteract plant oxidative bursts (Zhang et al., 2020b). The partial restoration of stress tolerance in complementary strains indicated the potential epistatic interactions or incomplete genetic rescue, warranting further transcriptomic analyses to map regulatory networks involving *Bd-SCP10*. In addition, the HOG pathway is associated with hyperosmotic stresses and the osmoregulation ability of fungal pathogens, primarily due to its involvement in an environment characterized by hyperosmotic stresses (Yaakoub et al., 2022). However, our studies found that mutants showed hypersensitivity in response to hyperosmotic stresses (0.5 M CaCl_2_, 1 M C_6_H_12_O_6_, and 1 M KCl, except for 1.5 M NaCl). Deletion of *Bd-SCP10* from Bd-wt has no link with the osmoregulation of 1.5 M NaCl, and we found the same results as mutant *Bdo*-*05381* from *B. dothidea* strains HTLW03 and ZY7 (Dong and Guo, 2020). These results indicate that *Bd-SCP10* is a positive modulator of the HOG pathway for the hyperosmotic stress response against 0.5 M CaCl_2_, 1 M C_6_H_12_O_6_, and 1 M KCl but a negative modulator for the hyperosmotic stress response against 1.5 M NaCl. Phylogenetically, *SCPs* are conserved in phytopathogenic fungi (Lv et al., 2023). However, *Bd-SCP10* clusters separately from *SCPs* homologs found in other fungi. These results suggest evolutionary divergence personalized to the host range or environmental niche of *B. dothidea*. This divergence may explain its unique regulatory roles, such as its apparent lack of relevance to NaCl tolerance. Comparative genomics could elucidate whether homologs of *Bd-SCP10* in related pathogens share similar functional specializations. While this study provides compelling evidence for multifunctionality, limitations exist for *Bd-SCP10*. The split marker knockout strategy although is reliable method however it does not rule out off-target effects. CRISPR-Cas9 validation could strengthen conclusions. Additionally, *in vitro*, fruit assays may not fully replicate field conditions, where host-pathogen interactions involve complex environmental and immunological variables. Future studies should aim to identify the direct substrates of *Bd-SCP10* through proteomic approaches to clarify its mechanistic role in host invasion. Host response profiling using transcriptomics or metabolomics could reveal how *Bd-SCP10* modulates plant defense pathways, particularly ROS detoxification and PR protein activity. In planta, validation under natural conditions will be essential to assess its role in systemic colonization and disease spread. Moreover, exploring *Bd-SCP10* associated stress regulatory networks and evolutionary comparisons with homologs of *SCPs* in related fungi may uncover unique adaptations. Finally, screening for specific inhibitors of *Bd-SCP10* could provide novel, eco-friendly strategies for controlling *B. dothidea* infections.

## 5 Conclusion

This study provides the first functional characterization of *Bd-SCP10*, a homolog of serine carboxypeptidase belonging to the S10 family in *B. dothidea*. Our results demonstrate that *Bd-SCP10* is essential for pathogenicity and stress tolerance, also revealing its role in maintaining growth and cell wall integrity. The complete loss of pathogenicity in mutants suggested that *Bd-SCP10* is an essential determinant of pathogenicity. However, identifying *Bd-SCP10* as a functional regulator of vegetative growth, pathogenicity, and stress tolerance, this work provides a foundation for developing eco-friendly control strategies against *B. dothidea* and related pathogens.

## Data Availability

The raw data supporting the conclusions of this article will be made available by the authors, without undue reservation.
