# Supplementary material for: Functional genome analysis reveals that serine carboxypeptidase Bd-SCP10 mediates vegetative growth, pathogenicity, and stress tolerance in Botryosphaeria dothidea
Source: Front Plant Sci. 2025 Nov 14;16:1678786. doi: 10.3389/fpls.2025.1678786 (PMC12661404; doi:10.3389/fpls.2025.1678786)
Supplement: Supplementary file 1 [file DataSheet1.doc]

**Supplementary Figures**

**
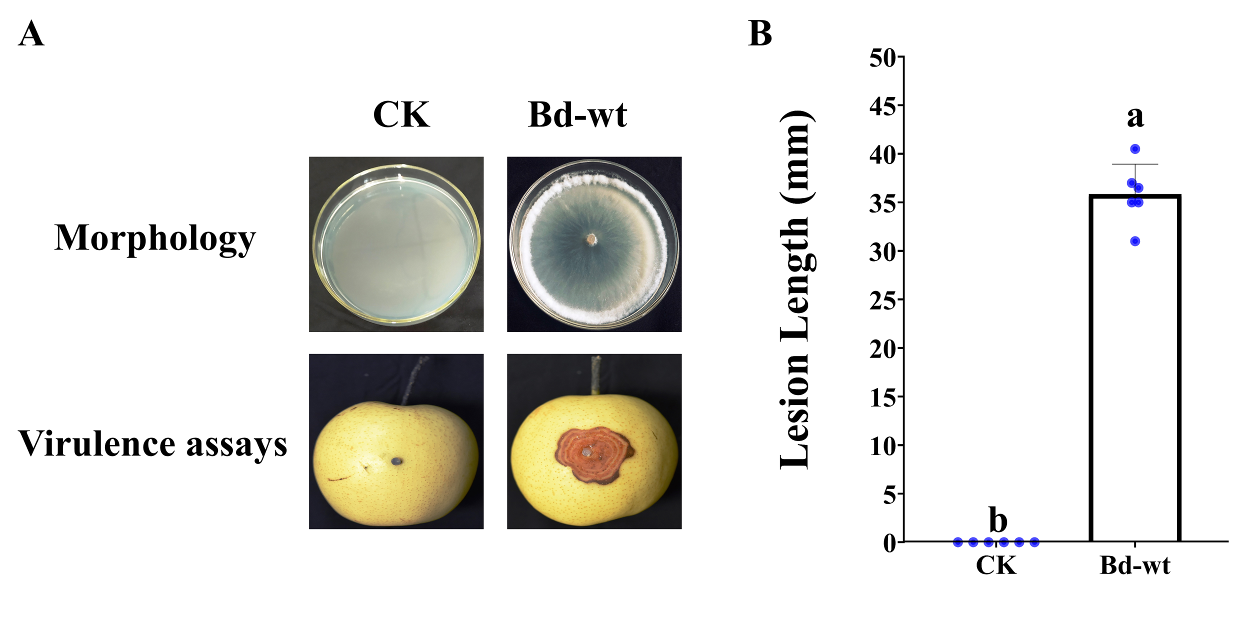
**

**Fig. S1.** Phenotypic and virulence assay. (A) Colony morphology of CK and Bd-wt on PDA at 5 d and 25°C, along with disease symptoms, appeared on pear fruits (var. huangguan) after 5 dpi at 25°C with 100% relative humidity. (B) The bar graph depicted the virulence analysis of lesion lengths, the alphabet on a column represented ANOVA with the LSD at *P≤0.05,* and the blue circular dots showed six replicates.


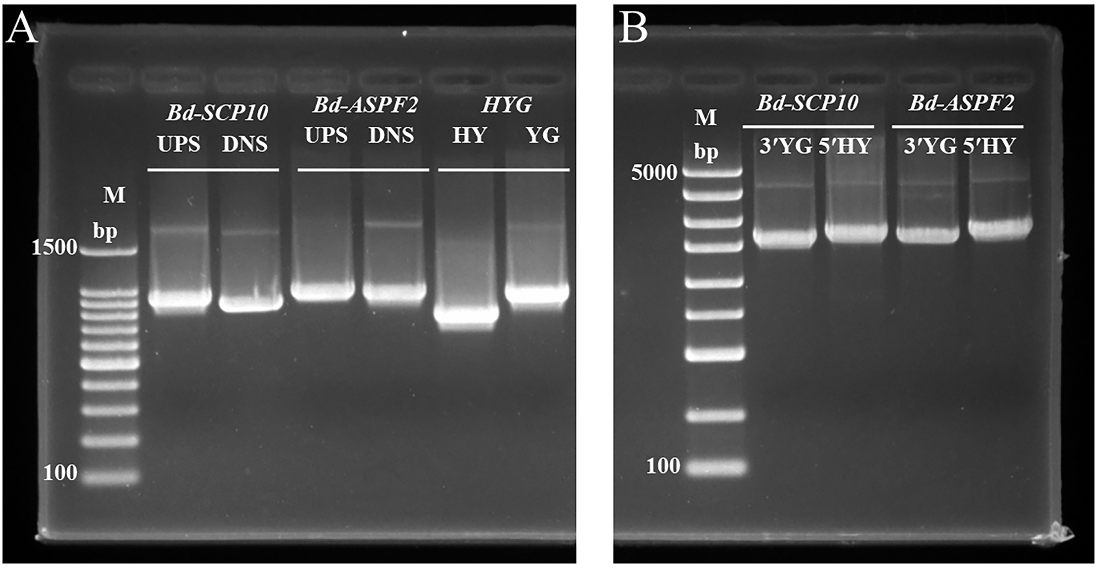


**Fig. S2.** Original gel documentation figures of *Bd-SCP10* gene knockout from Bd-wt through split marker strategy and PCR identification of the deletion mutants. (A) The upstream flanking (UPS, 887 bp) and downstream flanking (DNS, 789 bp) regions and parts of the HYG resistance cassette (HY, 767 bp and YG, 931 bp) were generated through first-round PCR. (Note: One gel was run for three samples, and *Bd-ASPF2* gene results are of no use regarding this article, as it is a different gene, and in Fig. 2, edited and added in the manuscript). (B) The UPS and DNS fused with HY and YG, resulting in 5′HY (1654 bp) and 3′YG (1720 bp) by fusion PCR, respectively. Here, M is denoted for DNA markers.


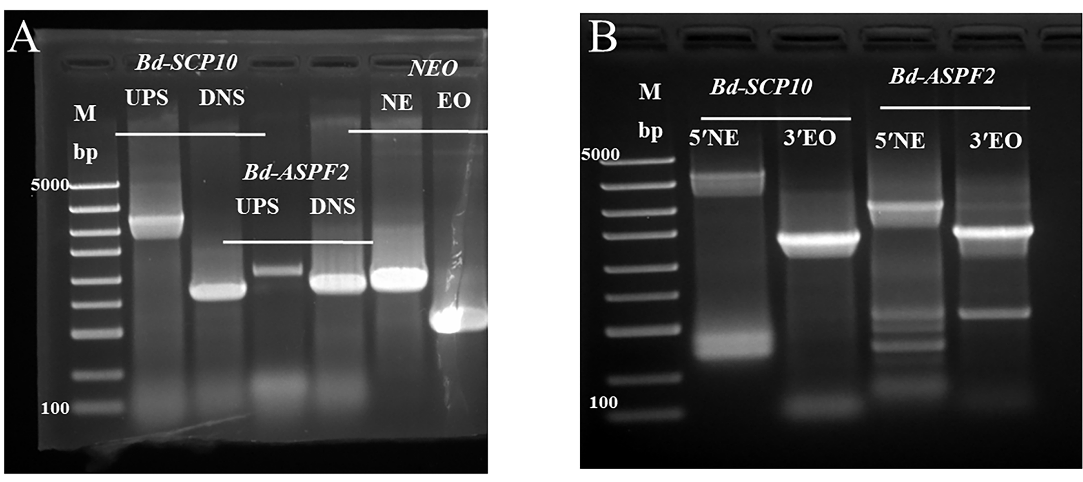


**Fig. S3.** Original gel documentation figures of *Bd-SCP10* gene Complementation in *ΔBd-SCP10*a through the split marker strategy and PCR identification of the complementary strains. (A) The UPS (2800 bp), DNS (832 bp), NE (975 bp), and EO (543 bp) parts of the NEO resistance cassette were generated by first-round PCR. (Note: One gel was run for three samples, and *Bd-ASPF2* gene results do not apply to this article, as they pertain to a different gene, which is addressed in Fig. 3, edited and added to the manuscript). (B) In fusion PCR, the fragments of first-round PCR (UPS and DNS) were used for fusion with fragments of NEO resistance cassette (NE and EO), and the final products were denoted as 5′NE (3775 bp) and 3′EO (1375 bp), respectively. Here, M is denoted for DNA markers.


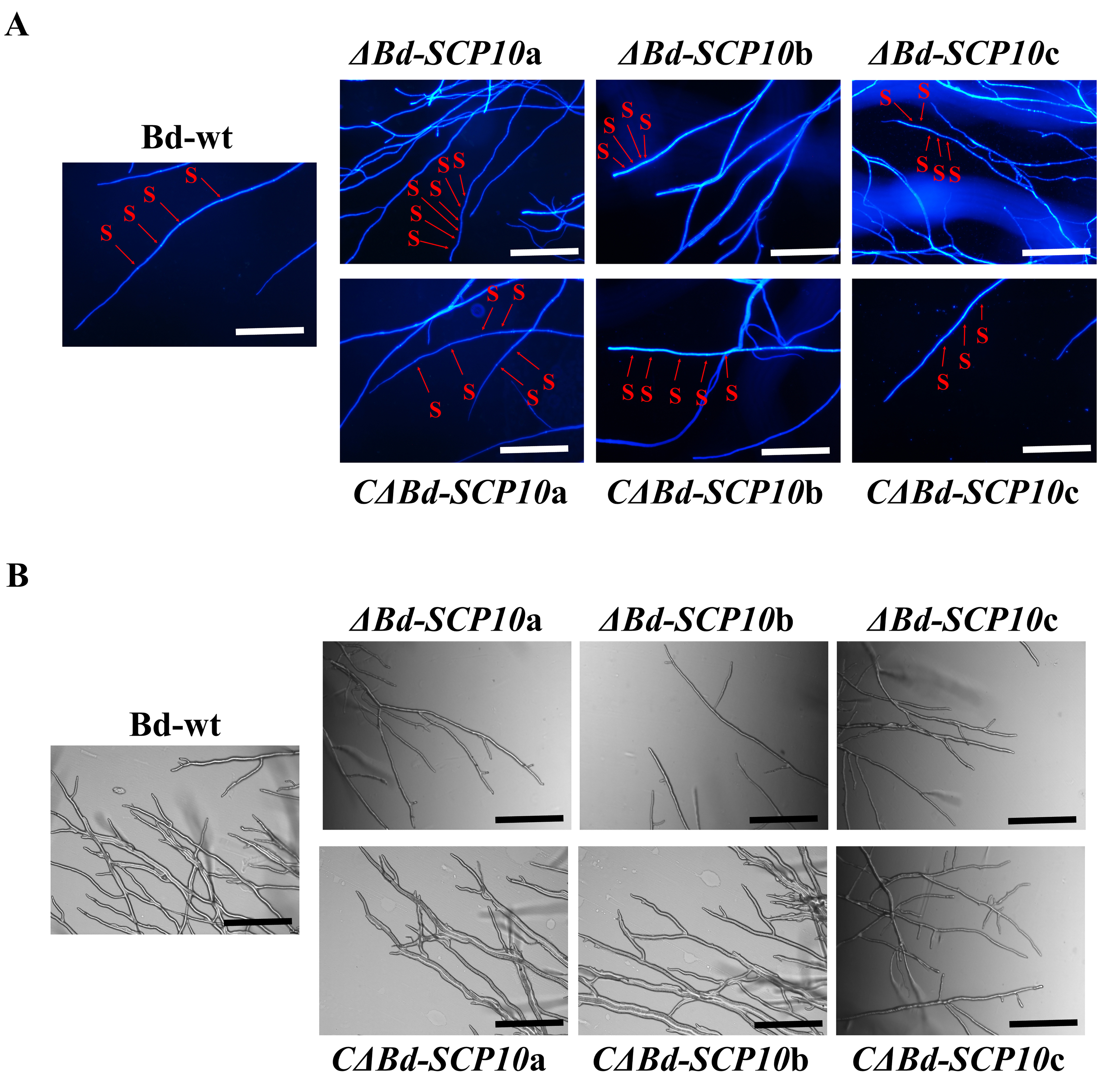


**Fig. S4.** Comparative analysis of vegetative growth and biomass production. (A) The fungal strains were grown on a glass slide cover and stained with Calcofluor-white (CFW), and microscopic analysis showed that the hyphal tips of Bd-wt and complemented strains have more extended septated cells than deletion mutants*,* which have more septation with short cell length at 10 µm (scale bar) and red-colored S with an arrow showing septation in hyphal tips. (B) Microscopic analysis revealed that deletion mutants have thinner mycelial growth than Bd-wt and complementary strains at 20µm (scale bar).

**Supplementary Tables**

**Table S1.** Primers used in this study.

| **Primer Name** | **Sequence (5' to 3')** |
| --- | --- |
| *Bd-SCP10*-F | GCCTGGACCCTGACATCTCG |
| *Bd-SCP10*-R | ATCCGTCCTTAATGGCTTGACT |
| *Bd-SCP10*-MUT-Up-F | AGGCCAATCACCACATCGAC |
| *Bd-SCP10*-MUT-Up-R | TTGACCTCCACTAGCTCCAGCCAAGCCCCTAACCGGCGTGATCCC |
| *Bd-SCP10*-MUT-Dn-F | GAATAGAGTAGATGCCGACCGCGGGTTCATGCCAGGGCCAACGAT |
| *Bd-SCP10*-MUT-Dn-R | GCAGGTATTCTGGTTGT |
| HYG-F | GGCTTGGCTGGAGCTAGTGGAGGTCAA |
| HYG-R | GAACCCGCGGTCGGCATCTACTCTATTC |
| YG-F | GATGTAGGAGGGCGTGGATATGTCCT |
| HY-R | GTATTGACCGATTCCTTGCGGTCCGAA |
| *Bd-SCP10-*COM-Up-F | GTGTGGGCGGTGTCGGGGGTGGGTGGAGGCCGCCGCCGCTGCCGTAGATGCACACCCGCGCCCT |
| *Bd-SCP10*-COM-Up-R | TGCTCCTTCAATATCATCTTCTGCCTTCTTCTTCCCCAAACCC |
| *Bd-SCP10*-COM-Dn-F | GAGTTCTTCTGAGGATCCACATGCCAGGGCCAACGAT |
| *Bd-SCP10*-COM-Dn-R | GCAGGTATTCTGGTTGT |
| NEO-F | CAGAAGATGATATTGAAGGAGCA |
| NEO-R | GTGGATCCTCAGAAGAACTC |
| EO-F | GGGAAGGGACTGGCTGCTATTG |
| NE-R | AAAAGCGGCCATTTTCCACCAT |
| *Bd-SCP10*-GENE-F | TGACATCTCGAACCGGAC |
| *Bd-SCP10*-GENE-R | TTCATCTTCCTGCGAGCC |

**Table S2. Comparative growth and biomass production analysis of Bd-wt, mutants, and complementary** strains.

| **Name of isolates** | **Growth rate (mm/day)** | **Rate of biomass production (g/day)** |
| --- | --- | --- |
| Bd-wt | 15.93±0.115a | 0.204±0.015ab |
| *ΔBd-SCP10*a | 2.333±0.088cd | 0.018±0.002c |
| *CBd-ΔSCP10*a | 15.40±0.173b | 0.210±0.004ab |
| *ΔBd-SCP10*b | 2.100±0.066d | 0.018±0.001c |
| *CBd-ΔSCP10*b | 15.46±0.251b | 0.200±0.005b |
| *ΔBd-SCP10*c | 2.422±0.117c | 0.019±0.002c |
| *CBd-ΔSCP10*c | 15.53±0.208b | 0.214±0.004a |

**Table S3. Infection rate of deletion mutants compared with Bd-wt, mutants, and complementary strains on pear fruits (var. huangguan).**

| **Name of isolates** | **Lesion length on fruits (mm)** |
| --- | --- |
| Bd-wt | 31.57±14.00a |
| *ΔBd-SCP10*a | 0.00±0.00c |
| *CBd-ΔSCP10*a | 28.71±12.98b |
| *ΔBd-SCP10*b | 0.00±0.00c |
| *CBd-ΔSCP10*b | 29.07±13.01b |
| *ΔBd-SCP10*c | 0.00±0.00c |
| *CBd-ΔSCP10*c | 28.35±12.61b |

**Table S4. Percentage growth inhibition of mutants due to stress response when growth medium amended with 0.04% SDS, 0.5 M CaCl_2_, and 1 M C₆H₁₂O₆ and comparative analysis with Bd-wt and complementary strains.**

| **Name of isolates** | **0.04% SDS** | **0.5 M CaCl_2_** | **1 M C₆H₁₂O₆** |
| --- | --- | --- | --- |
| Bd-wt | 27.60±3.34b | 7.53±1.11d | 5.01±1.06c |
| *ΔBd-SCP10*a | 47.61±1.11a | 43.70±4.12ab | 22.79±2.93a |
| *CBd-ΔSCP10*a | 33.93±6.16b | 8.65±3.14cd | 0.86±0.36d |
| *ΔBd-SCP10*b | 45.0±2.36a | 42.83±1.68b | 15.90±1.92b |
| *CBd-ΔSCP10*b | 29.72±2.46b | 14.26±3.67c | 2.15±0.97cd |
| *ΔBd-SCP10*c | 49.94±8.75a | 49.34±6.40a | 25.27±4.57a |
| *CBd-ΔSCP10*c | 29.38±4.08b | 13.92±2.43c | 1.92±1.09cd |

**Table S5. Percentage growth inhibition of mutants due to stress response when growth medium amended 1.5 M NaCl, 1 M KCl, and 0.05% H_2_O_2_ and comparative analysis with Bd-wt and complementary strains.**

| **Name of isolates** | **1.5 M NaCl** | **1 M KCl** | **0.05% H_2_O_2_** |
| --- | --- | --- | --- |
| Bd-wt | 75.31±0.83abc | 15.28±5.17c | 1.67±0.95c |
| *ΔBd-SCP10*a | 74.75±0.74abc | 44.75±1.12a | 59.00±2.72a |
| *CBd-ΔSCP10*a | 76.1±2.29ab | 30.07±3.06b | 0.86±0.36c |
| *ΔBd-SCP10*b | 72.41±3.30c | 42.28±7.62a | 46.02±2.75b |
| *CBd-ΔSCP10*b | 76.07±1.13ab | 22.84±0.89bc | 1.07±0.74c |
| *ΔBd-SCP10*c | 73.44±1.66bc | 48.02±5.29a | 58.15±6.10a |
| *CBd-ΔSCP10*c | 77.25±0.89a | 25.30±2.99b | 2.55±2.76c |
